# Graft-versus-host Reactions in the Rabbit

**DOI:** 10.1038/bjc.1960.8

**Published:** 1960-03

**Authors:** K. A. Porter

## Abstract

**Images:**


### GRAFT-VERSUS-HOST REACTIONS IN THE RABBIT

K. A. PORTER

*From the Department of Pathology, St. Mary's Hospital Medical School, London, W.2*

Received for publication December 1, 1959

UNTIL recently the homograft reaction was mainly studied from the aspect of rejection of the foreign tissue transplant by its host. Yet, as Medawar (1958) has pointed out, grafting is an act of parabiosis, however unequal the partners to the union may be, and the possibility of the transplant mounting a counter-attack against the recipient must be considered.

It was the work of Dempster (1953) and Simonsen (1953) on kidney homotransplants in dogs that first indicated that reactions of this nature might be more than a theoretical hazard. Since then the grave consequences of graft intolerance of the host have been recognised following induction of tolerance in newborn animals by the injection of adult spleen cells (Billingham and Brent, 1957 ; Simonsen, 1957), following treatment of lethally X-irradiated animals with homologous bone marrow (Trentin, 1956 ; Congdon and Urso 1957). and following injection of Fl hybrid mice with adult spleen cells from the parental strains (Cole and Ellis, 1958 ; Trentin, 1958 ; Gorer and Boyse, 1959). In all these instances the injected mice are for one reason or another unable to destroy the introduced foreign lymphoid cells which then proliferate and proceed to assail the host cells.

Although it is generally agreed that the victims lose weight and characteristically show atrophy of their lymphoid tissue, few detailed descriptions of the pathological appearances associated with these syndromes are available.

The purpose of this report therefore is to describe and compare the histological changes seen in rabbits suffering from secondary irradiation disease and runt disease.

### MATERIALS AND METHODS

*Animals.*.—Young adult Chinchilla rabbits which were not inbred in the genetic sense were used throughout this study.

*Production of radiation-chimaeras.*—Lethally X-irradiated rabbits were treated intravenously with bone marrow cells from normal adult rabbits.

1. *X-irradiation.*—Whole body X-irradiation was given as a horizontal beam to 2 animals at a time from a Westinghouse machine under the following conditions : 220 kV., 12·5 mA., 70 cm. target to skin distance, 1·0 mm. Cu and 1·0 mm. Al filters, H.V.L. 1·8 mm. Cu, dose rate to skin 33·4 r per minute, to centre of animal 21·1 r per minute. The dosage was divided ; an initial dose of 600 r at the centre of the animal was followed 24 hours later by 500 r, and after another 24 hours by a further 500 r, giving a total of 1600 r (L.D. 100/30 days).

2. *Bone marrow.*—A suspension of adult homologous bone marrow was prepared from a female rabbit as described earlier (Porter and Murray, 1958), and 1200 ($\times$ $10^6$) nucleated cells injected into the marginal ear vein of an irradiated male rabbit 1–3 hours following the last dose of X-rays, and within 1 hour of the death of the female donor.

3. *Criterion for success.*—The successful establishment of a radiation-chimaera was accepted only if female type heterophils (amphophils) appeared in the irradiated male rabbit (Porter, 1957*a*).

*Induction of immunological tolerance*

Spleen cells from adult homologous female rabbits were injected into foetal rabbits (Porter, 1960*a*).

1. *Preparation of spleen cell suspensions.*—A mature female rabbit weighing 2·5–3·0 kg. was anaesthetised with intravenous Nembutal and a splenectomy performed through an upper midline abdominal incision. Immediately after removal the spleen was finely chopped under aseptic conditions in heparanized saline solution and then suspended by gently drawing up and down through a wide bore cannula. After filtration through nylon bolting cloth of 90 $\mu$ porosity a cell count was made and a suitable quantity of the spleen suspension containing 50 ($\times 10^6$) nucleated cells injected into the recipient animal.

2. *Injection of foetal rabbits.*—A rabbit at the 20th or 22nd day of pregnancy (normal gestation period is 31 days), was anaesthetised with intravenous Nembutal and a midline abdominal incision made extending from the level of the last pair of nipples to just below the umbilicus. When the abdomen had been explored to determine the number of foetuses present, the free end of one horn of the uterus was delivered through the wound. The position of each foetus was easily seen and its head and back identified by palpation. Adult spleen tissue suspension was then injected through the wall of the uterus into the peritoneal cavity of each foetus. After returning the uterus to the abdomen by gentle steady pressure, the peritoneum and linea alba were sutured as one layer with 3–0 chromic gut. The skin was sutured separately.

3. *Skin grafts.*—When possible the recipients of spleen cell suspension were later challenged with skin grafts from the spleen donor. These were full thickness homografts 2 cm. in diameter sutured into prepared beds upon the ears as described by Stark, Brownlee and Grunwald (1958).

*Weight.*—The rabbits were weighed daily for the first month, then at weekly intervals.

*Antibiotic therapy.*—All irradiated rabbits were given tetracycline hydrochloride intramuscularly 50 mg. per day for the first 2 weeks following irradiation.

*Pathology.*—All animals that were killed or died were examined post mortem. A pencil of marrow was dissected from the right femur, the sample always being taken from a comparable position at the mid-point of the shaft. Most tissues were fixed in formol-saline, but the marrow, lymph nodes and spleen were fixed in Helly's fluid and all were routinely stained with haematoxylin and eosin. Special stains were used where necessary, and marrow smears were stained by Leishman's method.

Sex chromatin was also sought in lymphocytes obtained from the lymph nodes and spleen of some animals, using the method outlined by Riis (1957). The cell suspension to be examined was incubated for 24 hours in an homologous plasma coagulum and then appropriately fixed and stained. The disadvantages of this technique have already been discussed (Porter, 1960*b*).

*Experimental procedure*

In this experiment 4 groups of animals were studied.

Group I consisted of 5 normal adult non-irradiated rabbits and 14 baby rabbits of various ages. Animals from this group were killed at appropriate times for comparison with those that had died or been killed amongst the treated groups.

Group II was composed of 20 X-irradited rabbits given no subsequent treatment. They were examined histologically after death.

Group III consisted of 20 radiation-chimaeras with " secondary disease ".

In the accumulation of this group all successful radiation-chimaeras that were regaining the weight lost immediately after irradiation, were subjected to biopsy of an inguinal or axillary lymph node at 14 days after marrow treatment. After this time any animal that continued to show a constant percentage of polymorphs with female sex chromatin, and that was clinically free of infection, but started to lose weight again, was regarded as developing " secondary disease ". Taking the day wasting was first noticed as the beginning of " secondary disease ", groups of 3 animals were killed on the 1st, 7th, 14th and 21st day of the process for histological examination. The remainder were examined whenever they happened to die.

Group IV was composed of 20 non-irradiated tolerant rabbits with " runt disease ".

In allocating animals to this group those rabbits subjected to intra-uterine homologous spleen injection that failed to gain weight properly after birth, were considered to have " runt disease ". The majority died aged 2–6 weeks and were then examined histologically. In addition groups of 2 animals were killed and examined at intervals of 1, 2 and 3 weeks after birth.

### RESULTS

*Rabbits exposed to lethal X-irradiation without marrow treatment* (*Group II*)

All rabbits in this group had died by the 10th day, the mean survival time being $8{\cdot}1 \pm 1{\cdot}12$ days. At post mortem some slight wasting was always present and the fur often appeared dull and tended to be shed easily.

*Bone marrow.*—There was destruction of practically all the haemopoietic cells of the femoral bone marrow, only the reticular cells surviving.

*Spleen.*—This organ was smaller than normal. The mean splenic weight being $0{\cdot}22 \pm 0{\cdot}062$ g. per kg. of body weight compared with $0{\cdot}55 \pm 0{\cdot}046$ g. for a group of 20 normal rabbits. The decrease in size was due to almost complete destruction of the lymphoid tissue. The reticular cells in the white pulp remained. The red pulp was often congested and always contained haemosiderin, much within macrophages.

*Lymphoid tissue elsewhere.*—The thymus showed destruction of cortical lymphocytes with shrinkage of the lobules and contraction of the stroma to form a solid sheet of cells in the region where the cortex had been. Similar destruction of lymphocytes with no evidence of regeneration was seen in the mesenteric, axillary and inguinal lymph nodes, and in the normal lymphoid collections in the gut, particularly the appendix. In 8 cases many red cells were present in the lymph node sinuses.

*Respiratory system.*—A necrotic and haemorrhagic non-purulent infection of the lungs by *Ps. pyocyaneus*, killed 5 of the animals in this group. Eight animals showed intra-alveolar haemorrhage without infection.

*Gastro-intestinal tract.*—Petechial haemorrhages into stomach and elsewhere in the gut were seen in 16 animals. Rupture of the fundus of the stomach caused the death of 4 animals and massive haemorrhage into the colon the death of 4 more. In 3 rabbits that died before 7 days there was a partial denudation of the intestinal mucosa ; the intervening areas consisting of flattened villi covered by stretched epithelial cells. In the remaining rabbits, regeneration was complete by the time they died from infection or haemorrhage.

*Liver.*—In 6 cases there was some atrophy of the liver cells and distension of the sinusoids with blood ; 5 more animals showed changes usually associated with chronic venous congestion.

*Testes.*—Active spermatogenesis had ceased.

No other characteristic lesions were noticed.

*Radiation-chimaeras with " secondary disease " (Group III)*

The animals with successful marrow transplants initially lost weight, but this was followed by recovery from about the 10th day after irradiation. However, in those developing " secondary disease ", at any time between the 16th and 40th days weight loss recurred, accompanied by early signs of diarrhoea. These symptoms became steadily more severe until the animal died. At post mortem the rabbits were invariably emaciated. Their fur was dull, dirty and shed easily.

*Bone marrow.*—All the animals, at whatever stage of " secondary disease " they were examined, showed a well-repopulated bone marrow (Fig. 1). Smears revealed the presence of " drumsticks " in some of the cells indicating that repopulation was from donor sources. Animals killed or dying after the 14th day of " secondary disease " showed loss of normal fat and increased cellularity. The longer the disease had been present, the more pronounced this hyperplasia, which was predominately of the myeloid series. Plasma cells were rarely seen in the bone marrow. Erythropoiesis and megakaryocyte formation appeared to be normal. A few phagocytic cells containing haemosiderin were always to be seen. In 12 of the animals the stroma consisted of mucoid material which stained positively with mucicarmine.

*Spleen.*—First day of " secondary disease ". In the 3 animals killed at the beginning of " secondary disease ", the spleens appeared slightly larger than normal. Microscopically there was good repopulation of the white pulp with lymphocytes, immature plasma cells and some transitional cells, using these terms as defined by Fagraeus (1948). Mitotic figures were frequent, and small germinal centres were present in 2 of the animals. The red pulp contained about as much haemosiderin as was present in the control irradiated animals of Group II.

Seventh day of " secondary disease ". In animals killed at this stage the spleens were enlarged, the mean weight being $1\cdot00 \pm 0\cdot183$ g. per kg. of body weight (normal $= 0\cdot55 \pm 0\cdot046$ g.), and microscopically the white pulp contained many transitional cells, immature plasma cells and a few mature plasma cells and lymphocytes (Fig. 2). Again mitotic figures were common. In 1 of the animals these cells were also found diffusely scattered throughout the red pulp. The congested red pulp contained plenty of haemosiderin, mostly in macrophages. Erythrophagocytosis was present in all 3 spleens.

*Fourteenth day of " secondary disease ".* The 3 rabbits killed at this time still showed slightly enlarged spleens with a mean weight of $0{\cdot}58 \pm 0{\cdot}03$ g. per kg. of body weight. Microscopically in some of the lymphoid nodules were cells with pyknotic or fragmented nuclei, and others which had undergone complete necrosis. Of the surviving cells a high proportion were mature plasma cells (Fig. 3). Other lymphoid nodules in the white pulp had already been severely depleted of cells and partly replaced by masses of smudgy fibrinoid material (Fig. 4). In one of the animals foreign body giant cells were associated with this fibrinoid substance. The red pulp was congested and now appeared to contain more haemosiderin than was present in the spleens of the control irradiated animals.

*Twenty-first day of " secondary disease ".* The spleens at this stage appeared shrunken with a mean weight of $0{\cdot}29 \pm 0{\cdot}03$ g. per kg. of body weight. Microscopically in 1 animal the white pulp had largely been replaced by immature collagen and numerous foreign body giant cells (Fig. 5). Lymphocytes, transitional cells and immature plasma cells had disappeared, but the red pulp contained scattered groups of mature plasma cells. Other animals examined at this time showed similar loss of lymphoid tissue and substitution of fibrous scars for Malpighian bodies, but without giant cells (Fig. 6). Scattered groups of mature plasma cells were, however, always present and in 2 animals small haemorrhages were also seen.

Two rabbits that died 30 and 34 days after the onset of " secondary disease " both showed extensive replacement of the depleted white pulp with mature collagen. In all instances the red pulp contained much haemosiderin.

*Other lymphoid tissue.*—When biopsied at 14 days after bone marrow treatment, the inguinal or axillary lymph nodes of those radiation-chimaeras which subsequently developed " secondary disease ", were small and showed early incomplete repopulation with lymphocytes (Fig. 7). In cell suspensions from a few of these lymph nodes female sex chromatin was demonstrated in lymphocytes by the method of Riis (1957).

By the time the first animals with " secondary disease " were killed the mesenteric and other lymph nodes were enlarged and showed an extensive proliferation of transitional cells and immature plasma cells, with relatively few mature plasma cells and lymphocytes. Lymphoid tissue elsewhere showed similar changes, but the thymus tended to lag in this process.

Little change was seen in the histological picture obtained at the 7th day of " secondary disease ", but by the 14th day many of the cells in the repopulated lymph nodes were undergoing necrosis (Fig. 8) and mature plasma cells were beginning to predominate amongst the surviving cells (Fig. 9). By the 21st day the lymph nodes were shrunken, depleted of lymphocytes, and only contained a sprinkling of mature plasma cells (Fig. 10). Lymphoid tissue elsewhere was similarly atrophied.

*Respiratory system.*—In 6 of the 8 radiation-chimaeras that perished from " secondary disease " the immediate cause of death was a patchy purulent peribronchiolar pneumonic consolidation. In 5 of these the organism responsible was *Ps. pyocyaneus.*

*Gastro-intestinal tract.*—Of the 20 radiation-chimaeras with " secondary disease " 4 animals showed discrete gastric ulceration. These chronic ulcers were about 0·5 cm. in diameter and situated at the pylorus. The high incidence of this lesion, often accompanied by perforation, is well recognised in the rabbit after large doses of X-irradiation (Porter, 1957*b*). A colitis was present in 6 animals. This was most severe in the descending colon.

*Liver.*—Occasional focal areas of necrosis were present in the liver lobules of 4 of the 8 animals that died, and in those of 2 of the animals killed at 14 days and 1 of the animals killed at 21 days. These lesions were scattered throughout the organ and were not consistently found in any one zone of the liver lobule. They did not seem to be related to coccidial infection. Two of the 20 animals with " secondary disease " showed biliary cirrhosis. This lesion has been described previously (Porter, 1960*d*) and is usually associated with recrudescence of infection with *E. stiedae.*

*Testes.*—Atrophy of these organs was usually present.

Amyloid was not found in these animals and no other characteristic lesions were noticed. The skin appeared normal.

*Rabbits suffering from " runt disease " (Group IV).*

All the animals in this group failed to gain weight at the normal rate. The severity of the disease varied greatly, so that whereas a few of the baby rabbits died within 15 days of birth following an " acute " attack, others suffered from a " chronic " attack causing great retardation of growth and death 50–70 days after birth. The mean survival time of those dying naturally was 31·93 $\pm$ 12·79 days.

Three runts were skin grafted from the spleen donor and proved to be fully tolerant for as long as they lived.

*Bone marrow.*—Of the 14 animals that died from " runt disease ", 3 showed an aplastic bone marrow with loss of most of the haemopoietic cells (Fig. 11 and 12) and 5 a femoral marrow greatly depleted of cells (Fig. 13). These changes were accompanied by clinical evidence of immune haemolysis of the animals' own red cells and a steep fall in the leucocyte and platelet counts (Porter, 1960*c*). The other 6 animals showed in 2 cases a bone marrow of normal cellularity and in 4 a granulocytic hyperplasia. Three of the rabbits with normal or hyperplastic bone marrow were males and smears showed occasional heterophils with " drumsticks ", and similar female cells were found in the peripheral blood, showing that some at least of the bone marrow cells were derived from the spleen donor.

Of the 6 animals killed at various times after birth, the 2 examined at one week both showed a bone marrow of normal cellularity in which female cells could not be found ; the 2 rabbits killed at 2 weeks showed loss of cellularity of the marrow ; and the marrow of 1 animal examined at 3 weeks was aplastic, whilst the other showed masses of donor type heterophils and their precursors.

*Spleen.*—In all 14 animals with " runt disease " that died naturally, the spleen appeared normal size in 8, and small in the remainder. Microscopically there was great diminution in the number of lymphoid cells in the splenic nodules and white pulp generally (Fig. 14 and 15). At best only a small halo of lymphocytes and mature plasma cells was left around the splenic arterioles ; and in 6 animals the Malpighian bodies had been completely replaced by immature or mature collagen (Fig. 16). The red pulp was congested in many, and erythrophagocytosis was prominent in all spleens. Extramedullary haemopoiesis, although present in some, was never very marked.

One of the rabbits killed a week after birth showed apparently normal lymphoid nodules in the spleen, but the other animal had an enlarged spleen with many transitional cells and some immature plasma cells in the white pulp. Cell suspensions from these spleens were examined by the method of Riis (1957), and some mononuclears with female sex chromatin found.

---

### EXPLANATION OF PLATES

FIG. 1.—Bone marrow from a rabbit killed 14 days after the onset of " secondary disease ". The marrow remains well repopulated, but there is loss of fat. Smears showed female cells. H. and E. × 110.

FIG. 2.—Spleen from a rabbit killed 7 days after the onset of " secondary disease ". The lymphoid nodule is well repopulated with transitional cells, immature plasma cells and a few lymphocytes. H. and E. × 110.

FIG. 3.—Spleen from a rabbit killed 14 days after the onset of " secondary disease ". Many of the cells in the lymphoid nodule are necrotic. H. and E. × 50.

FIG. 4.—Spleen from a rabbit killed 14 days after the onset of " secondary disease ". The lymphoid nodule is depleted of cells and partly replaced by masses of smudgy fibrinoid material. H. and E. × 50.

FIG. 5.—Spleen from a rabbit killed 21 days after the onset of " secondary disease ". The white pulp has been replaced by immature collagen and numerous foreign body giant cells. H. & E. × 50.

FIG. 6.—Spleen from a rabbit killed 21 days after the onset of " secondary disease ". The lymphoid tissue has disappeared and its place taken by fibrous tissue and a few scattered plasma cells. H. and E. × 50.

FIG. 7.—Inguinal lymph node removed at biopsy from a rabbit 14 days after irradiation and marrow treatment. There is incomplete repopulation with lymphocytes. Female sex chromatin was demonstrated in some of the cells from this node. H. and E. × 50.

FIG. 8.—Inguinal lymph node removed at post mortem from a rabbit killed 14 days after the onset of " secondary disease ". Note that many of the lymphoid cells have undergone necrosis. A biopsy of a node from the other groin of this animal, before the onset of " secondary disease ", is shown in Fig. 7. H. and E. × 50.

FIG. 9.—Higher magnification of part of a mesenteric lymph node from a rabbit killed 14 days after the onset of " secondary disease ". Mature plasma cells predominate amongst the surviving cells. H. and E. × 400.

FIG. 10.—Mesenteric lymph node from a rabbit killed 21 days after the onset of " secondary disease ". Lymphocytes have disappeared, only reticular cells and a sprinkling of mature plasma cells remain. H. and E. × 50.

FIG. 11.—Femoral bone marrow from a normal baby rabbit 30 days old for comparison with Fig. 12. H. and E. × 110.

Fig. 12.—Bone marrow from a baby rabbit that died from " runt disease " when 30 days old. There is almost complete aplasia of haemopoietic cells. Compare with Fig. 11 which shows the normal appearance in a rabbit of this age. H. and E. × 110.

FIG. 13.—Marrow from a rabbit that died from " runt disease " when 40 days old. There is great depletion of haemopoietic cells when compared with the marrow shown in Fig. 11. H. and E. × 110.

FIG. 14.—Spleen from a normal baby rabbit 21 days old for comparison with Fig. 15. H. and E. × 50.

FIG. 15.—Spleen from a rabbit with " runt disease ", killed at 21 days. The lymphoid nodule contains very few lymphocytes. Compare with Fig. 14 which shows the normal appearence in a rabbit of this age. H. and E. × 50.

FIG. 16.—Spleen from a rabbit that died from " runt disease " when 70 days old. There is loss of lymphoid tissue and replacement by fibrous tissue. H. and E. × 140.

FIG. 17.—Mesenteric lymph node from a normal baby rabbit 20 days old for comparison with Fig. 18. H. and E. × 50.

FIG. 18.—Mesenteric lymph node from a rabbit that died from " runt disease " when 20 days old. Lymphocytes have disappeared, but reticular cells and mature plasma cells remain. Compare with Fig. 17 which shows the normal appearance in a rabbit of this age. H. and E. × 50.

FIG. 19.—Appendix from a normal baby rabbit 21 days old for comparison with Fig. 20. H. and E. × 50.

FIG. 20.—Appendix from a rabbit with " runt disease " killed at 21 days. There is almost complete loss of lymphoid tissue when compared with Fig. 19 which shows the normal for this age. H. and E. × 50.

1

2

3

4

Porter.

5

6

7

8

9

10

11

12

Porter.

13

14

15

16

17

18

19

20

The spleens of the animals killed at 2 weeks were normal size, but both microscopically showed proliferation of transitional and immature plasma cells in the white pulp with few lymphocytes and 1 rabbit showed in addition small patches of fibrinoid necrosis in the lymphoid nodules.

The 2 animals killed at 3 weeks both had small spleens with great reduction in lymphoid content of the white pulp and in 1 of the rabbits the splenic nodules had been replaced by collagen.

*Lymphoid tissue elsewhere.*—All the animals that died from " runt disease " showed, compared with controls of the same ages, shrunken lymph nodes containing very few lymphocytes (Fig. 17 and 18). This loss of lymphocytes was also a striking feature of the intestinal lymphoid tissue, e.g., Peyer's patches, and was most obvious in the appendix (Fig. 19 and 20). The thymus was however, an exception, for although there was undoubtedly some decrease in lymphocytes, this was nowhere so obvious as in lymphoid tissue elsewhere.

In the animals killed at intervals after birth, primitive cells, including transitional and immature plasma cells, were numerous in the lymph nodes at 1 and 2 weeks, but by 3 weeks the nodes were atrophic. Lymphocytes were at all times scarce.

*Respiratory system.*—The lungs of 12 of the 14 animals that died from " runt disease " showed bronchopneumonic lesions at post mortem. In the 8 animals with an aplastic or hypoplastic bone marrow this was a necrotic and haemorrhagic non-purulent infection, whilst in the remainder it took the form of a purulent peri-bronchiolar consolidation.

*Gastro-intestinal tract.*—Apart from an ulcerative enteritis in 3 animals, no other specific lesions were seen.

*Liver.*—In the livers of 6 of the 14 animals that died, and in the liver of 1 of the animals killed at 3 weeks, there were small focal areas of necrosis. These lesions were identical with those seen in the irradiated rabbits suffering from " secondary disease ". Evidence of coccidiosis was sought but not found.

No renal or vascular lesions were observed in any of the animals. The skin and other organs appeared normal. Amyloid was not found in any of the rabbits.

### DISCUSSION

As the present study shows the pathological changes in " runt disease " are very like those seen in " secondary disease ". In both cases the animals become progressively more sick, waste and often develop diarrhoea. Death from infection usually occurs some 2–6 weeks after the onset of the process. In both instances by using sex chromatin as a biological marker it has been shown that the foreign cells injected, or their descendants, persist and are present in the lymph nodes and spleen, i.e., these animals are cellular chimaeras. At first free proliferation rapidly repopulates the host's lymphoid system with poorly differentiated pyronin-positive donor cells resembling the transitional and immature plasma cells described by Fagraeus (1948). After this initial increase there is a generalised regression with atrophy of the new lymphoid tissue and its gradual replacement by collagen and scattered collections of mature plasma cells. Focal liver necroses, which have been discussed previously (Porter, 1960*b*), are also a feature of both syndromes.

When the haemopoietic tissues are considered, however, certain differences are apparent between " runt disease " and " secondary disease ". In " secondary disease " there is rapid repopulation of the aplastic bone marrow, and even when the rabbit is dying this restored marrow always remains cellular and generally shows an extensive granulocytic hyperplasia. Use of the " drumstick " marker shows that this colonisation is from proliferation of donor cells, and this is confirmed by finding female type heterophils in the peripheral blood.

In " runt disease ", on the contrary, the bone marrow is frequently destroyed and the animal develops a severe anaemia. Only in a few runts is this destruction of host bone marrow accompanied by progressive replacement with haemopoietic cells of donor origin.

This difference is not difficult to understand when one remembers that in producing radiation-chimaeras a very large dose of haemopoietic cells and their precursors is given intravenously to an animal whose own bone marrow has been destroyed by X-rays ; whereas in producing runts spleen suspension containing relatively few such haemopoietic cells is given intraperitoneally to an animal whose own bone marrow is intact.

In both instances an immune haemolysis of host red cells is seen, accompanied by a raised indirect serum bilirubin, erythrophagocytosis and a positive Coomb's test. Also in both, as the disease progresses, the peripheral blood lymphocyte count fails steadily (Porter, 1960*c*).

These observations underline the essential similarity between " secondary disease " and " runt disease " in the rabbit. In both it seems the host is immunologically defenceless : in " secondary disease " because of irradiation damage, in " runt disease " because the immune system is insufficiently mature. The injected foreign cells proliferate, invade, and repopulate the lymphoid system of the host. Indirect evidence then suggests that they proceed to attack the host cells, but how, and whether this involves antibody, is not known. Further, why histological evidence of such an attack should be confined to haemopoietic, lymphoid and possibly hepatic tissue, is equally obscure. As the postulated assault continues, destruction of the restored lymphoid tissue also gradually occurs. At present, no explanation of this secondary loss of donor cells is entirely satisfactory. Kaplan and Rosston (1959) suggest that the foreign cells probably die in the course of killing host target cells : a process which they envisage may necessitate direct contact between each donor cell and a very few target cells. It may even be that the excessive amount of host antigenic material coming to the foreign lymphoid tissue simply overwhelms and exhausts it. However, it is important to be clear that no experimental precedent for such " exhaustion " exists. Whatever the exact mechanism, it does seem that death of the chimaera is the outcome of destruction of the antibody-producing cells of both host and donor.

As might be anticipated, giving irradiated rabbits homologous lymph node suspension as well as bone marrow leads to rapid repopulation of the host's spleen and lymph nodes with foreign cells and the onset of an " accelerated " form of " secondary disease " (Porter, 1960*b*).

Conversely, the incidence of " secondary disease " can be greatly reduced by using foetal haemopoietic cells, which are known to be immunologically immature, instead of adult bone marrow to produce the radiation-chimaeras (Uphoff, 1958 ; Porter, 1959.)

Other examples of graft-against-host reactions have recently been recognised and although the details of each syndrome vary according to the circumstances of the experiment, the end result is always wasting and lymphoid atrophy.

Thus, if splenic cells from parental strain donors are injected into F1 hybrid mice a wasting disease results which closely resembles " secondary disease " (Trentin, 1958). In this instance the foreign cells are not rejected because the F1 hybrid mice possess in their tissues all the antigens of both parent strains. The injected lymphoid cells therefore proliferate and attack the host producing histological changes very like those described in the present paper (Nowell and Cole, 1959).

Similarly, many rat pairs placed in parabiosis with a cross-circulation develop " parabiosis intoxication ", in which one animal remains well while the other rapidly wastes and his lymphoid tissue atrophies. When such homologous rats are separated after 5 days in parabiotic union a previously exchanged skin homograft may persist in an animal which is dying with general lymphoid atrophy and severe anaemia (Nakič and Silobrčič, 1958).

It thus seems that any transplantation of immunologically competent cells into an animal whose immune defences are for some reason paralysed, is potentially hazardous and may, under conditions favourable to the invading cells, produce a lethal wasting syndrome.

### SUMMARY

Secondary irradiation disease and " runt disease " in rabbits are described and compared.

Outstanding features of both are progressive wasting and diarrhoea, early splenomegaly and enlargement of lymph nodes, followed later by shrinkage of these organs and focal liver necroses. Overwhelming infection is usually the immediate cause of death.

With the help of sex chromatin and " drumstick " markers, it is shown that in both instances the animals are cellular chimaeras. There is early invasion and repopulation of host lymphoid tissues by proliferating donor-type transitional cells and immature plasma cells. Later, these cells undergo necrosis and this is sometimes associated with fibrinoid changes and foreign body giant cells. The end result is extreme lymphoid atrophy with fibrosis.

The only histological difference between the two conditions is that in " secondary disease " the aplastic bone marrow is rapidly colonised with donor cells and is still highly cellular when the animal dies, whereas in " runt disease " the host's bone marrow is frequently destroyed and less often is there repopulation with donor-type haemopoietic cells.

The similarity between these syndromes and those known as " parabiosis intoxication " and F1 hybrid " wasting disease " is noted and the conclusion reached that they are all graft-against-host reactions.

The author wishes to thank Miss Jane Rendall for valuable technical assistance, Dr. M. Hulbert and Sister Woodward of the Radiotherapy Department, St. Mary's Hospital, for providing the X-irradiation facilities, and Lederle Laboratories Division, American Cyanamid Company, Pearl River, New York, for generous supplies of tetracycline hydrochloride.

This work was supported by a grant from the Medical Research Council.

### REFERENCES

Billingham, R. E. and Brent, L.—(1957) *Transplant. Bull.*, **4**, 67.
Cole, L. J. and Ellis, M. E.—(1958) *Science*, **128**, 32.
Congdon, C. C. and Urso, I. S.—(1957) *Amer. J. Path.*, **33**, 749.
Dempster, W. J.—(1953) *Brit. J. Surg.*, **40**, 447.
Fagraeus, A. (1948) *Acta med. scand.*, Suppl. 204.
Gorer, P. A. and Boyse, E. A.—(1959) *Immunology*, **2**, 182.
Kaplan, H. S. and Rosston, B. H.—(1959) *Stanf. med. Bull.*, **17**, 77.
Medawar, P. B.—(1958) *Proc. Roy. Soc.* B., **148**, 145.
Nakič, B. and Silobrčič, V.—(1958) *Nature, Lond.*, **182**, 264.
Nowell, P. C. and Cole, L. J.—(1959) *Transplant. Bull.*, **6**, 435.
Porter, K. A.—(1957*a*) *Ibid.*, **4**, 129.—(1957*b*) *Brit. J. exp. Path.*, **38**, 401.—(1959) *Ibid.*, **40**, 273.—(1960*a*) *Nature, Lond.* **185**, 789.—(1960*b*) *Clin. Radiol.*, **11**, 22.—(1960*c*) *Ann. N.Y. Acad. Sci.* (in press).—(1960*d*) *Brit. J. exp. Path.* **41**, 72.
*Idem* and Murray, J. E.—(1958) *J. nat. Cancer Inst.*, **20**, 189.
Riis, P.—(1957) *Nature, Lond.*, **179**, 785.
Simonsen, M.—(1953) *Acta path. microbiol. scand.*, **32**, 36.—(1957) *Ibid.*, **40**, 480.
Stark, R. B., Brownlee, H. and Grunwald, R. P.—(1958) *Ann. N.Y. Acad. Sci.*, **73**, 772.
Trentin, J. J.—(1956) *Proc. Soc. exp. Biol. N.Y.*, **92**, 688.—(1958) *Ann. N.Y. Acad. Sci.*, **73**, 799.
Uphoff, D. E.—(1958) *J. nat. Cancer Inst.*, **20**, 625.


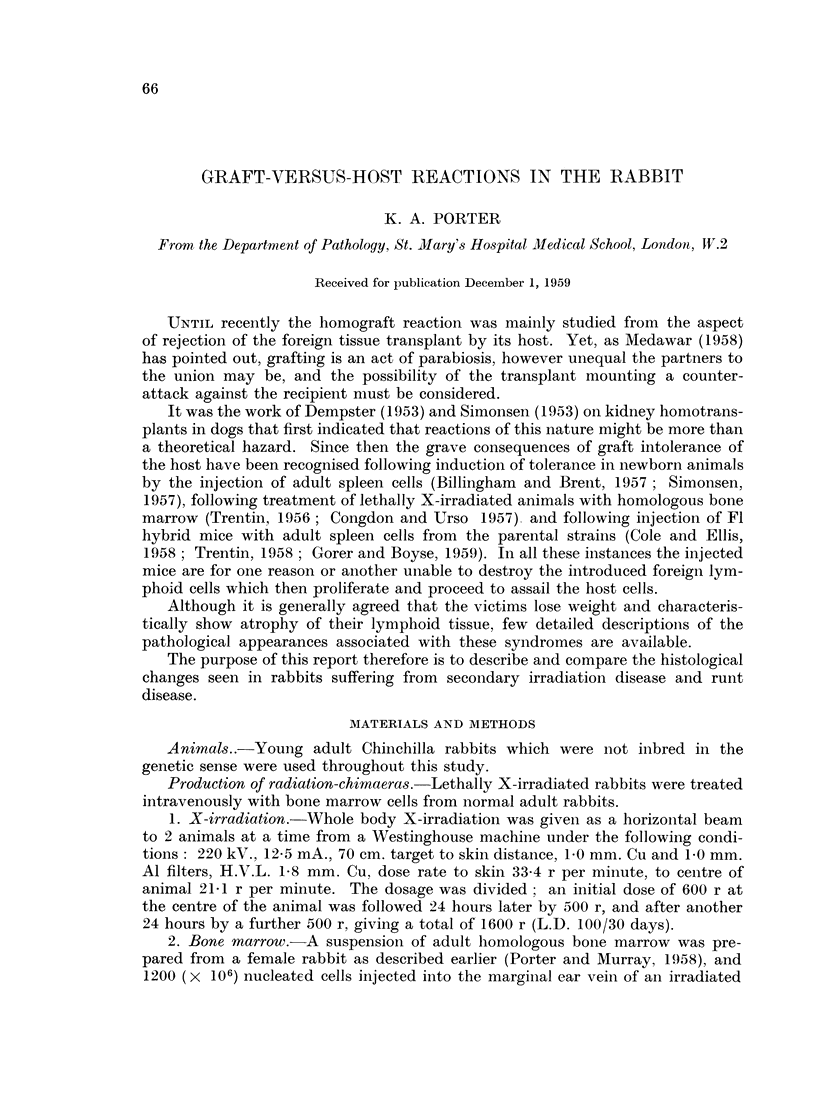

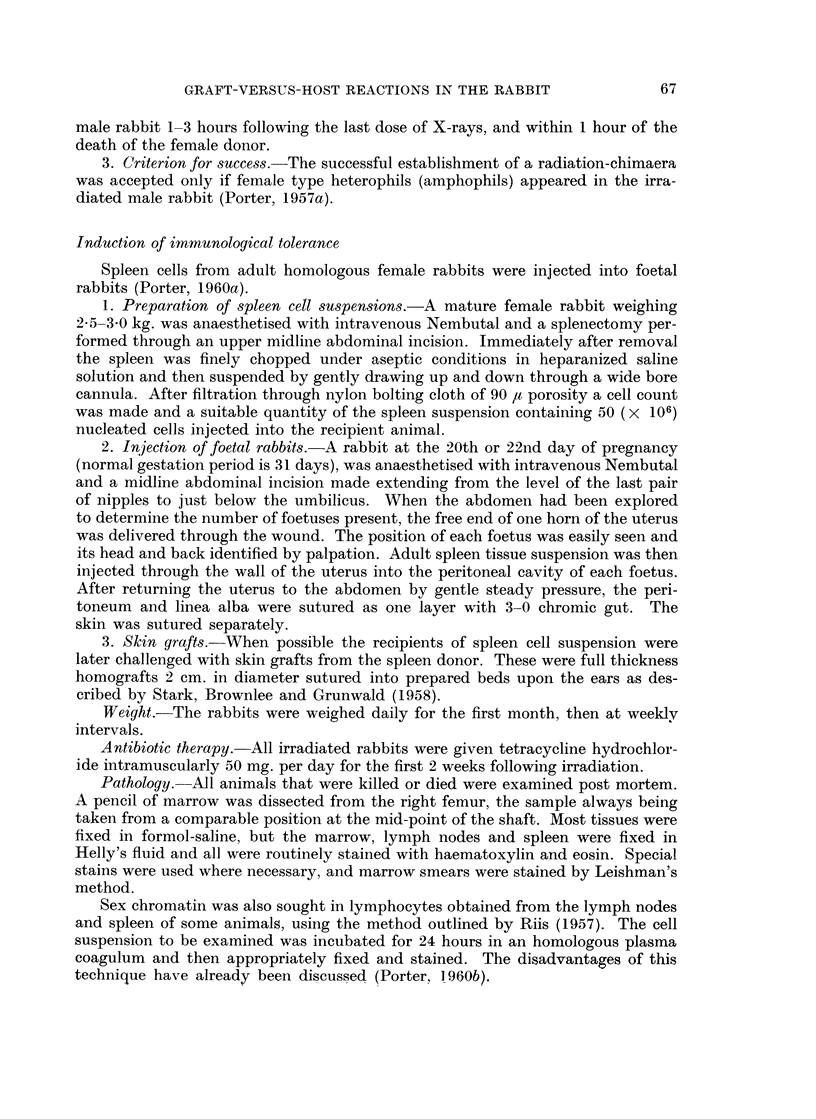

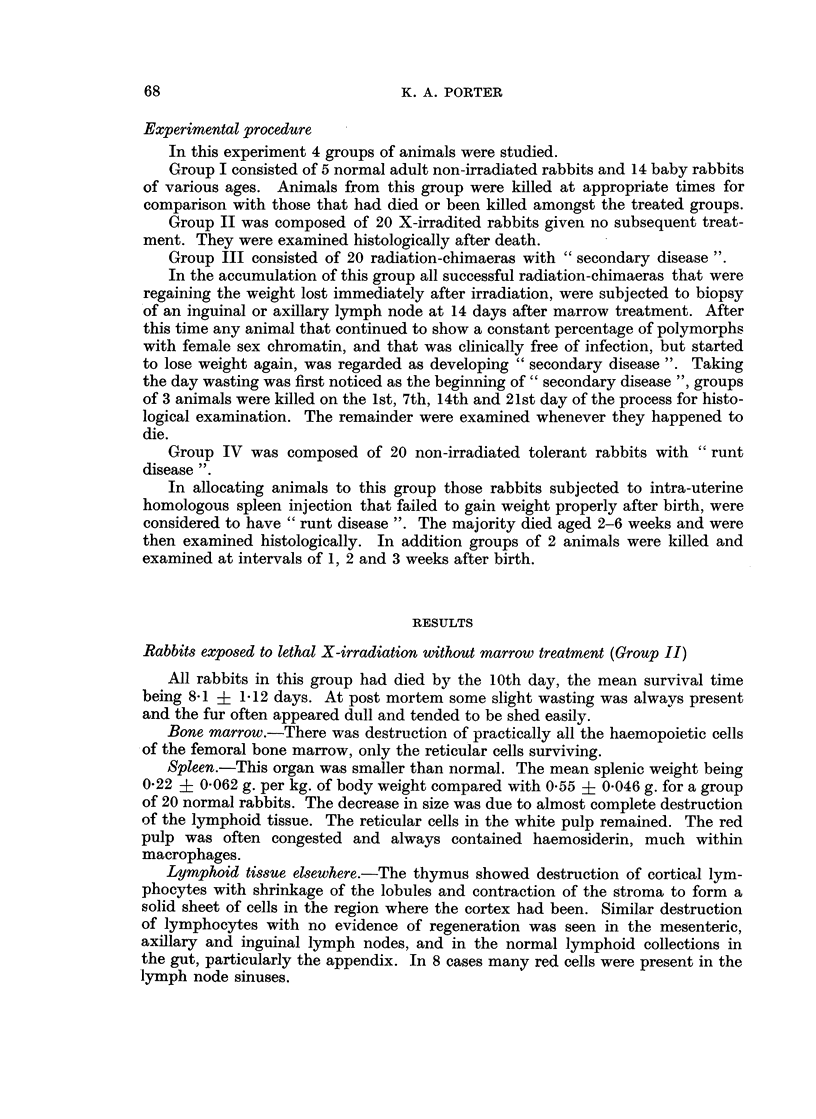

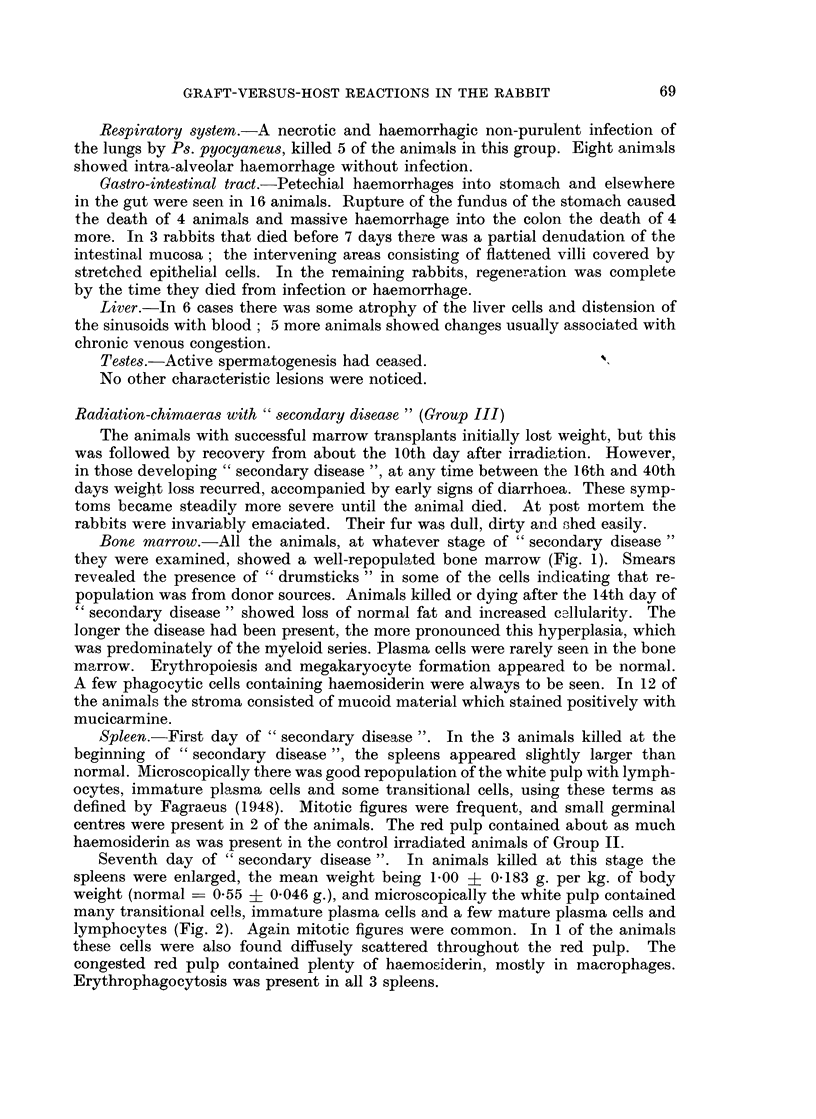

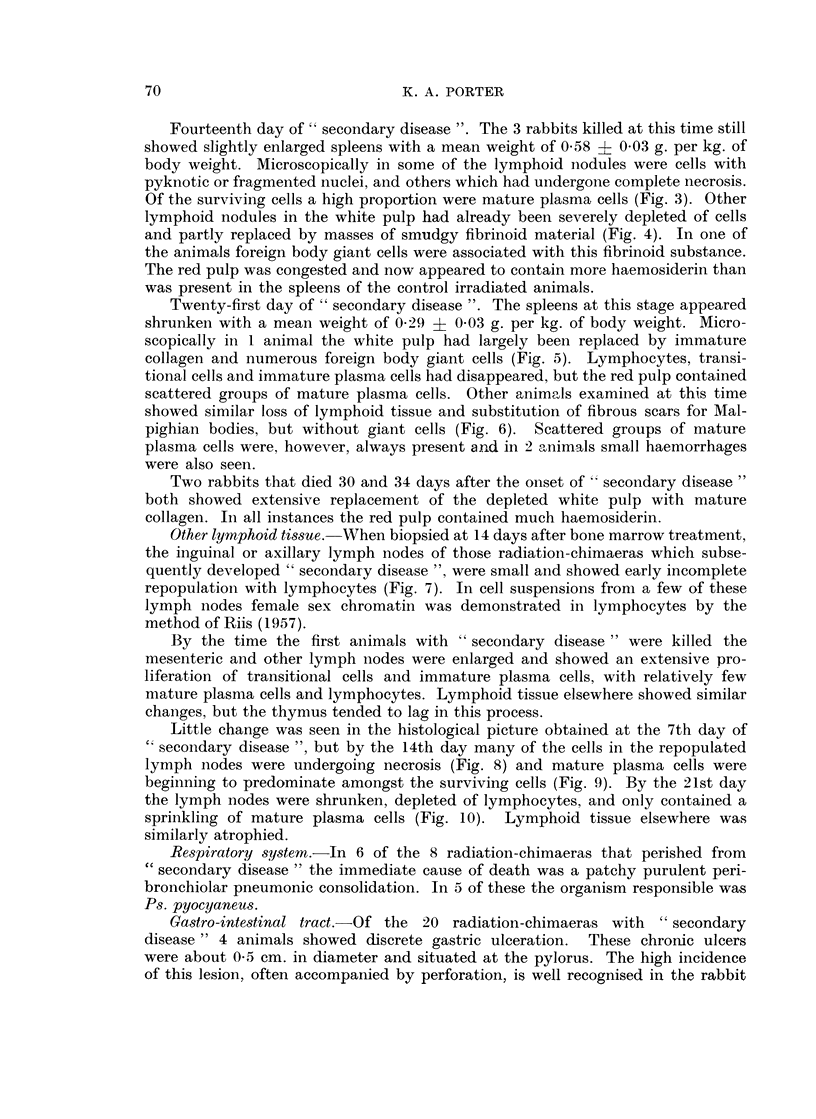

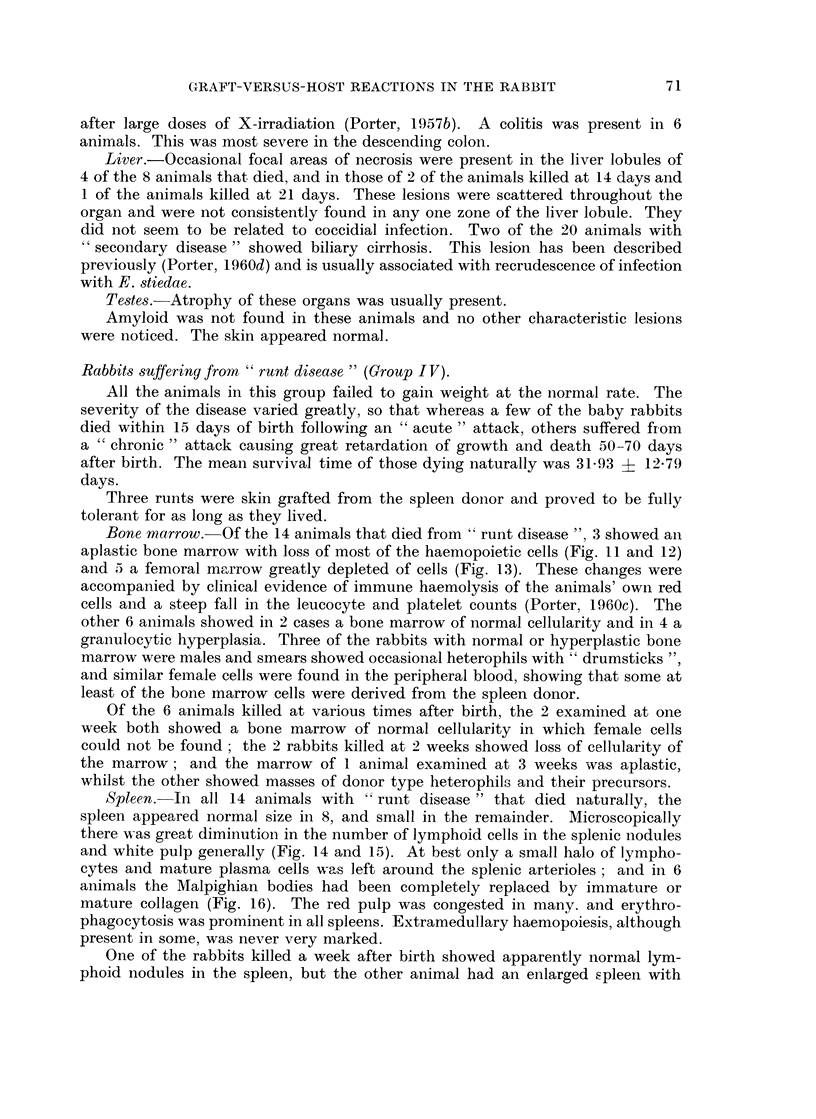

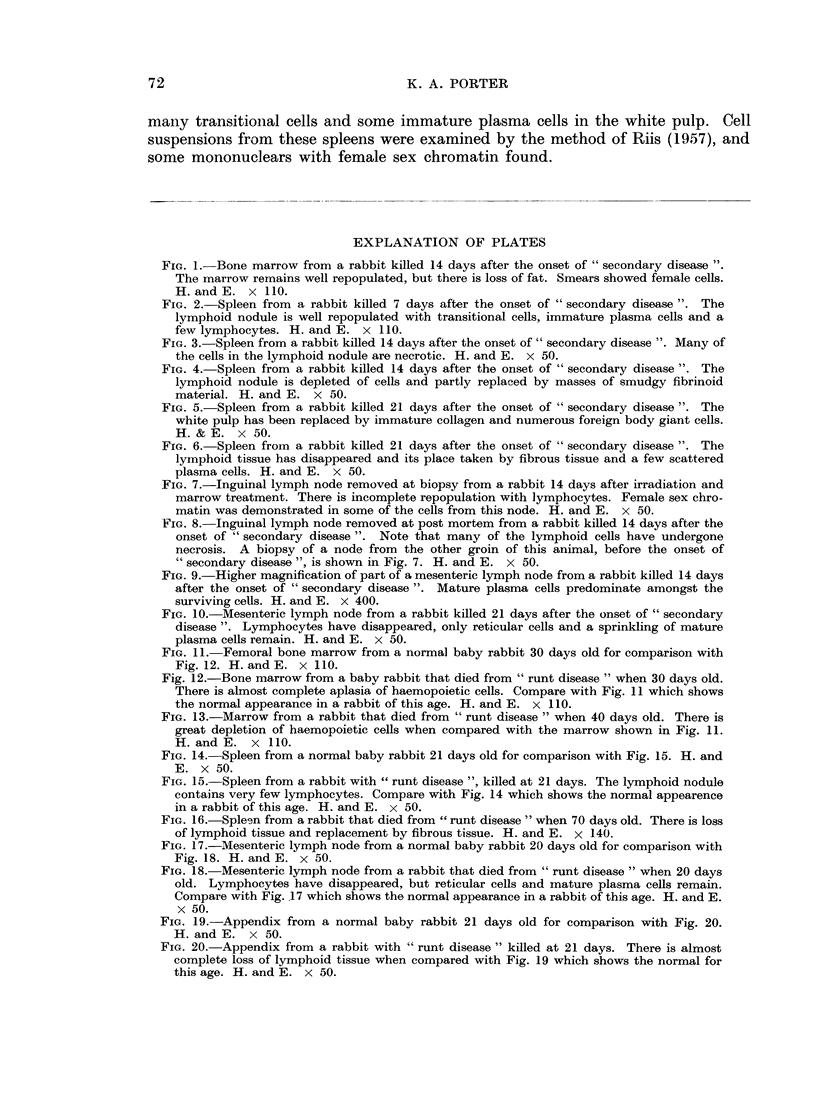

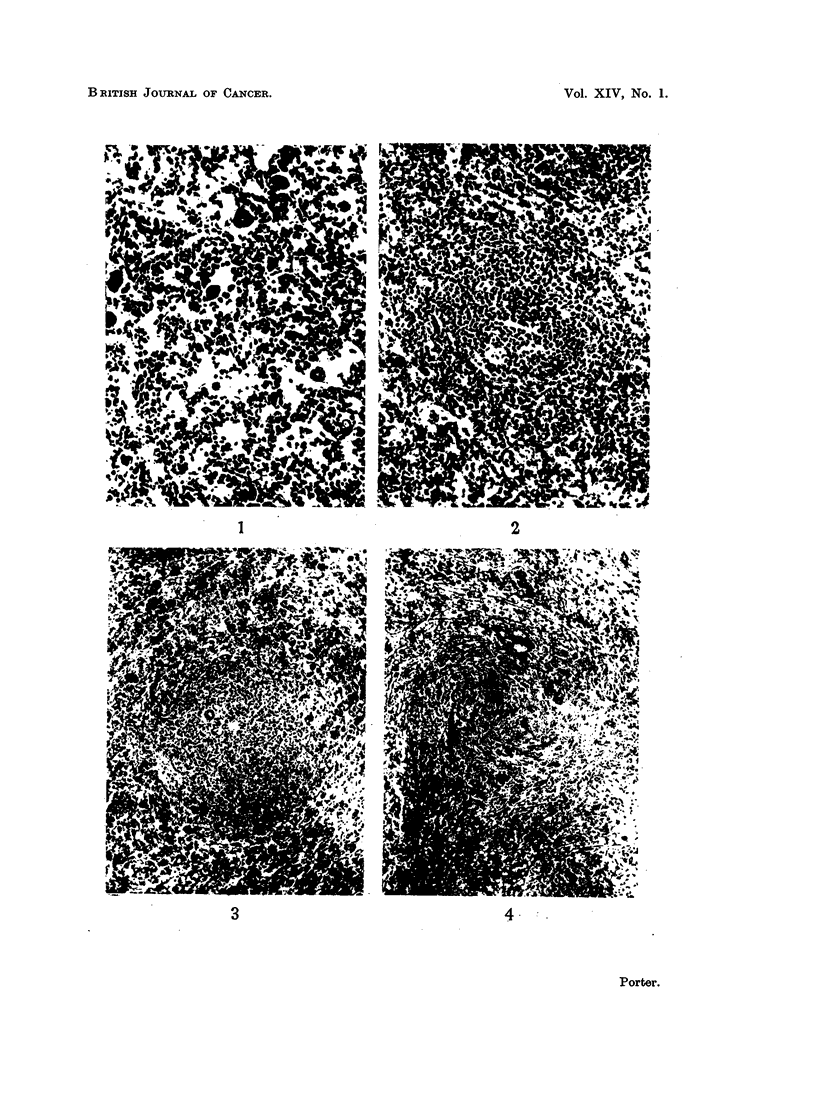

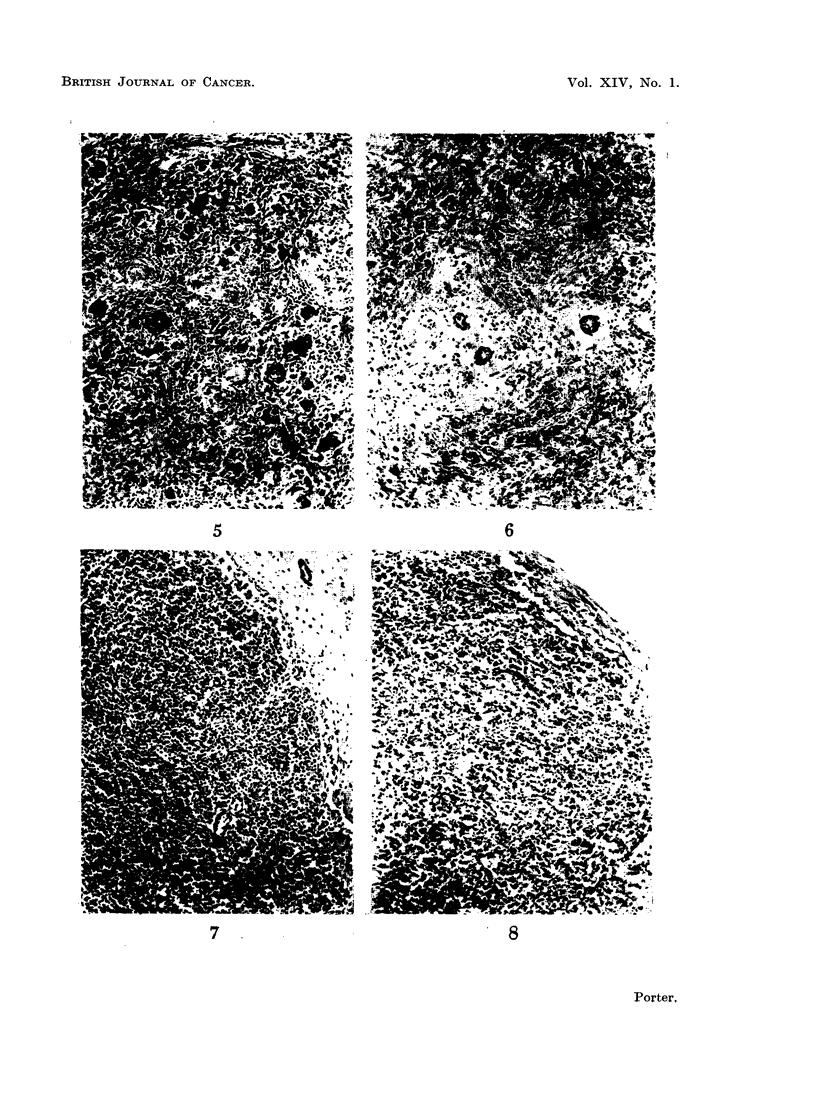

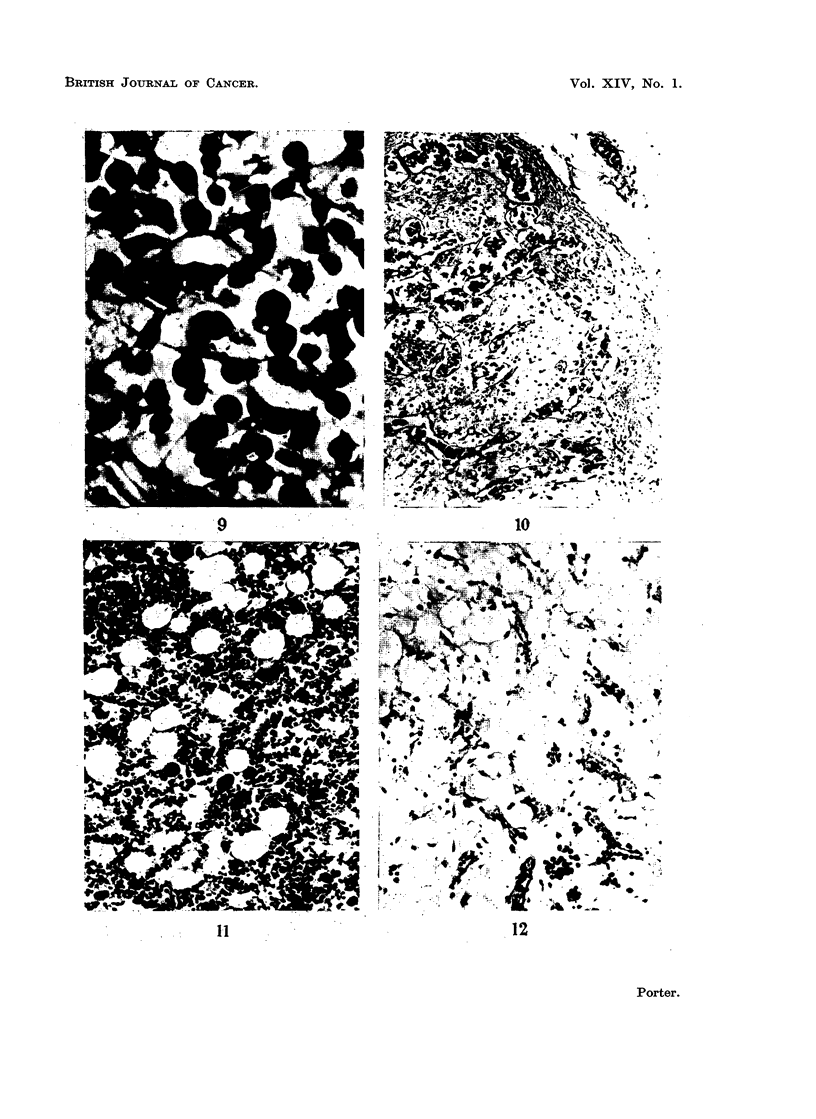

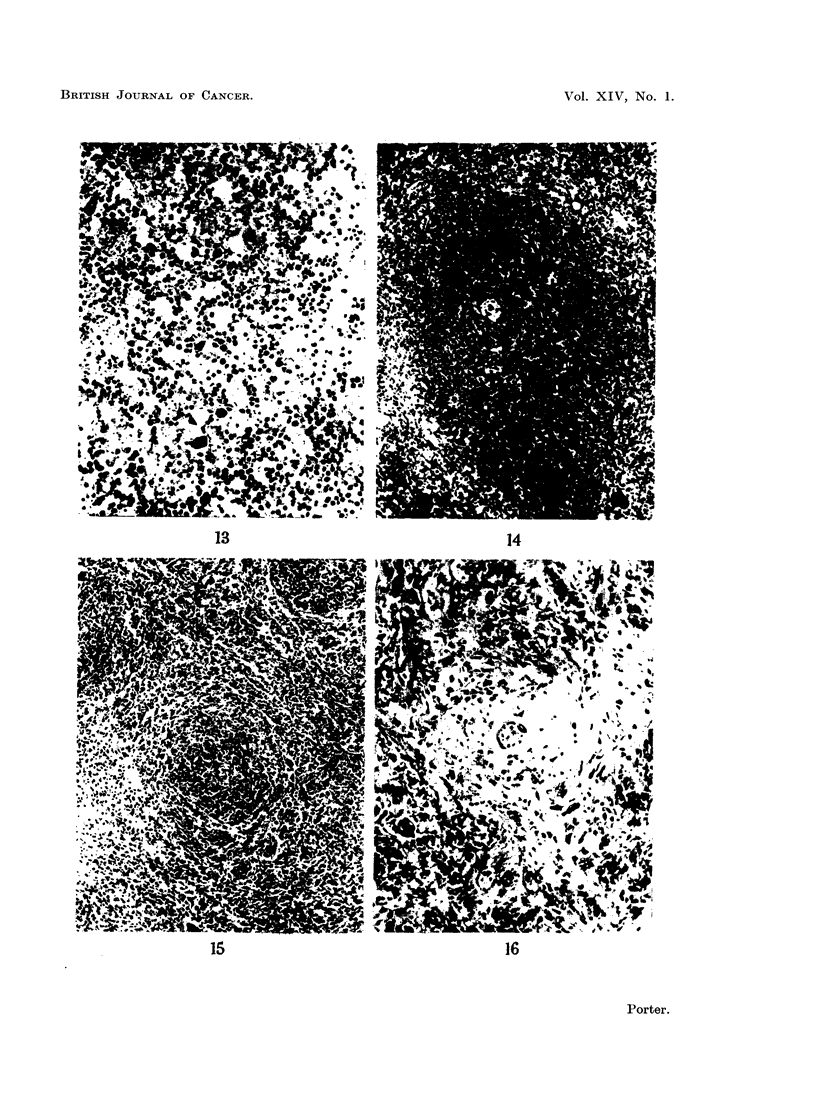

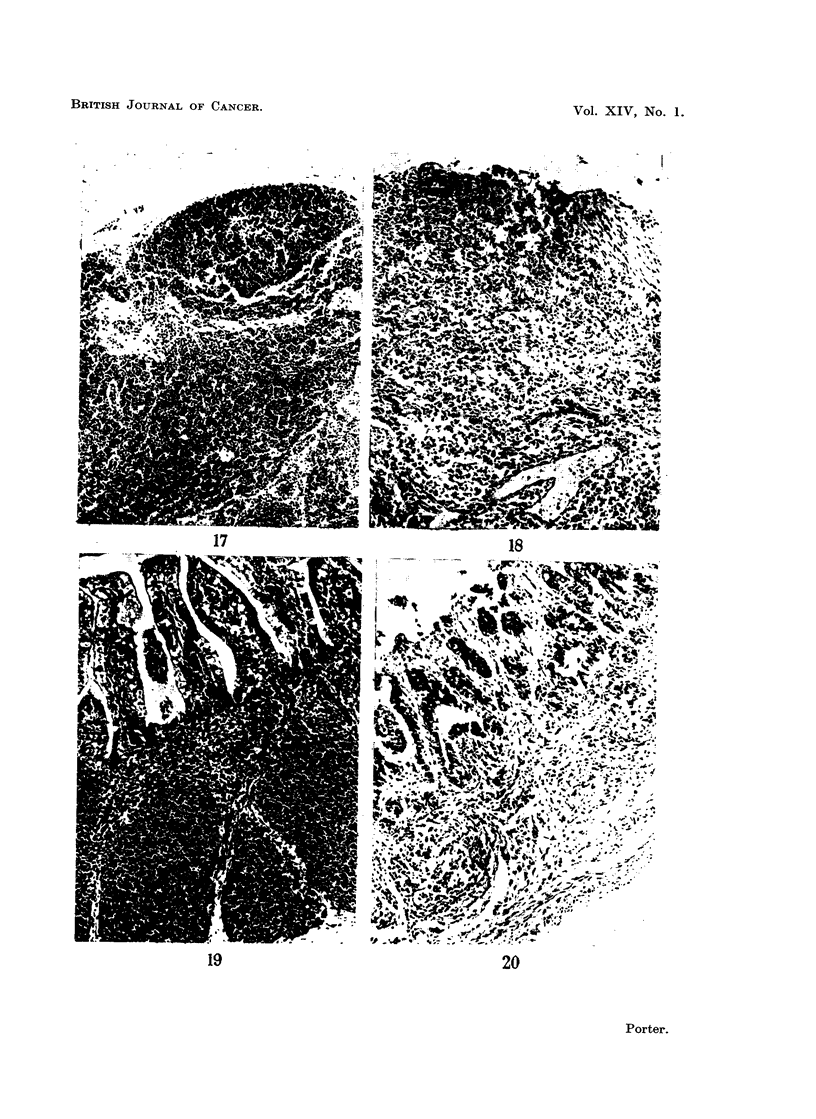

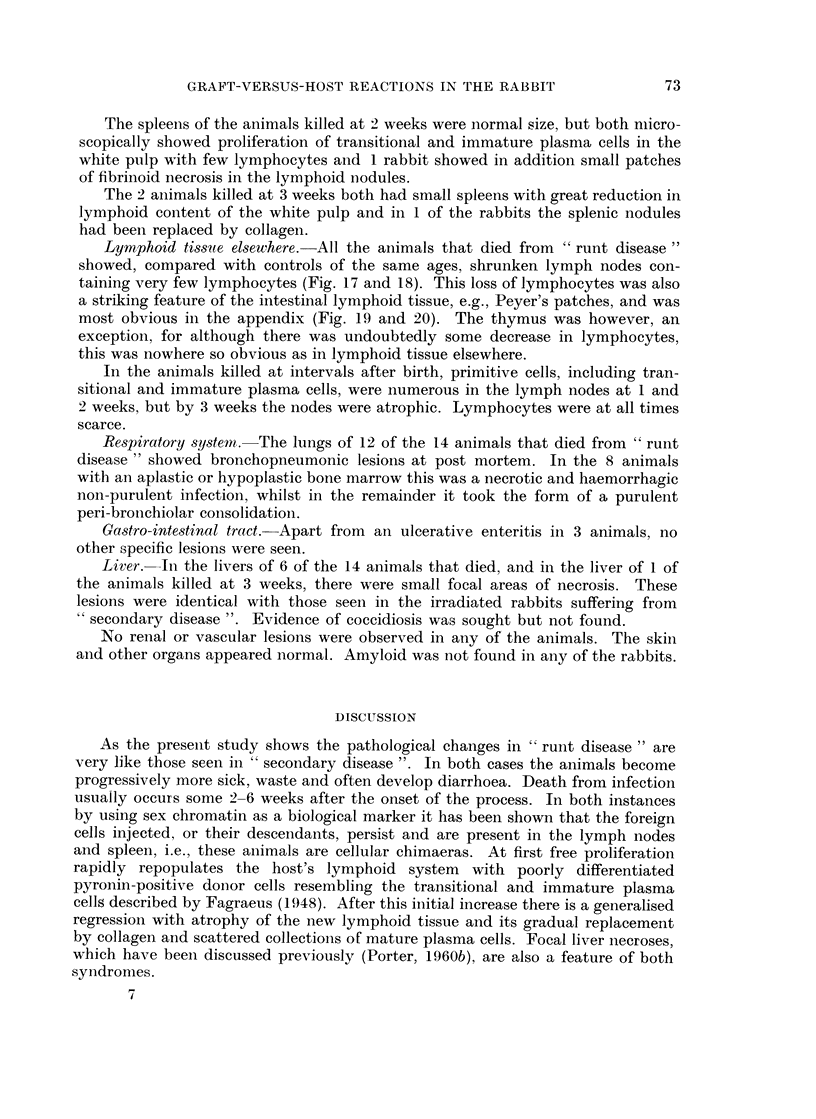

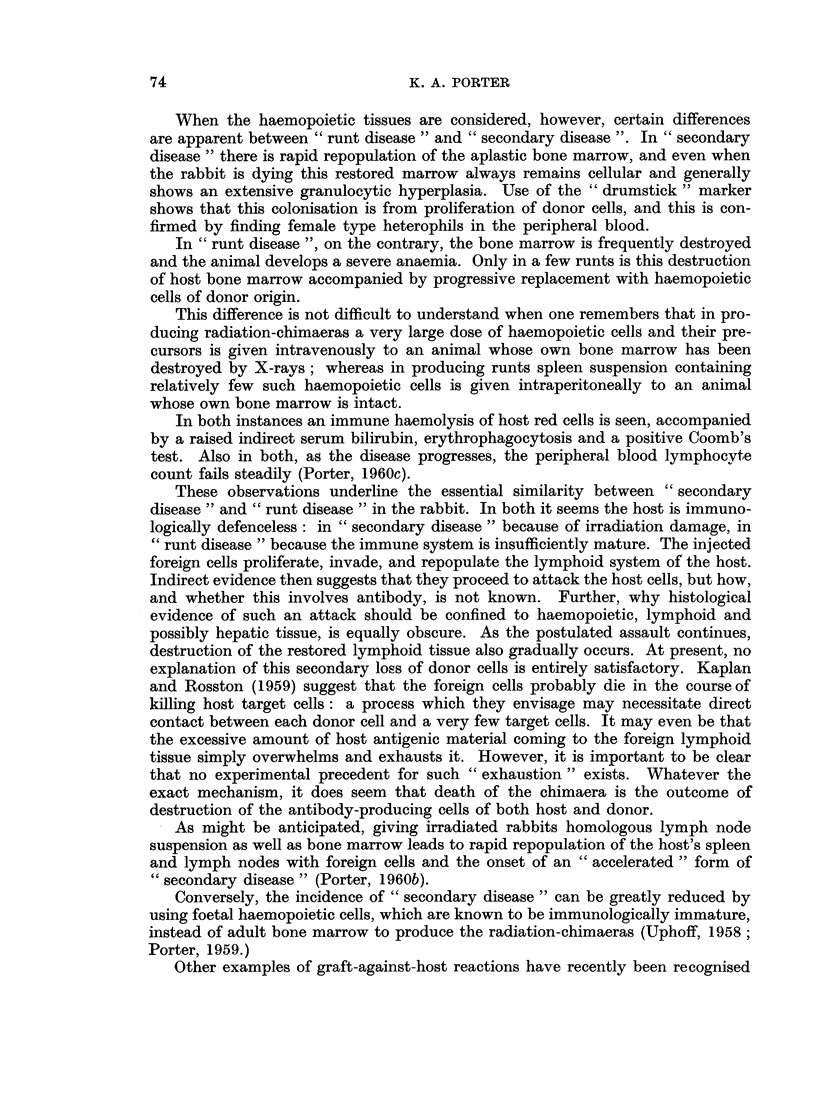

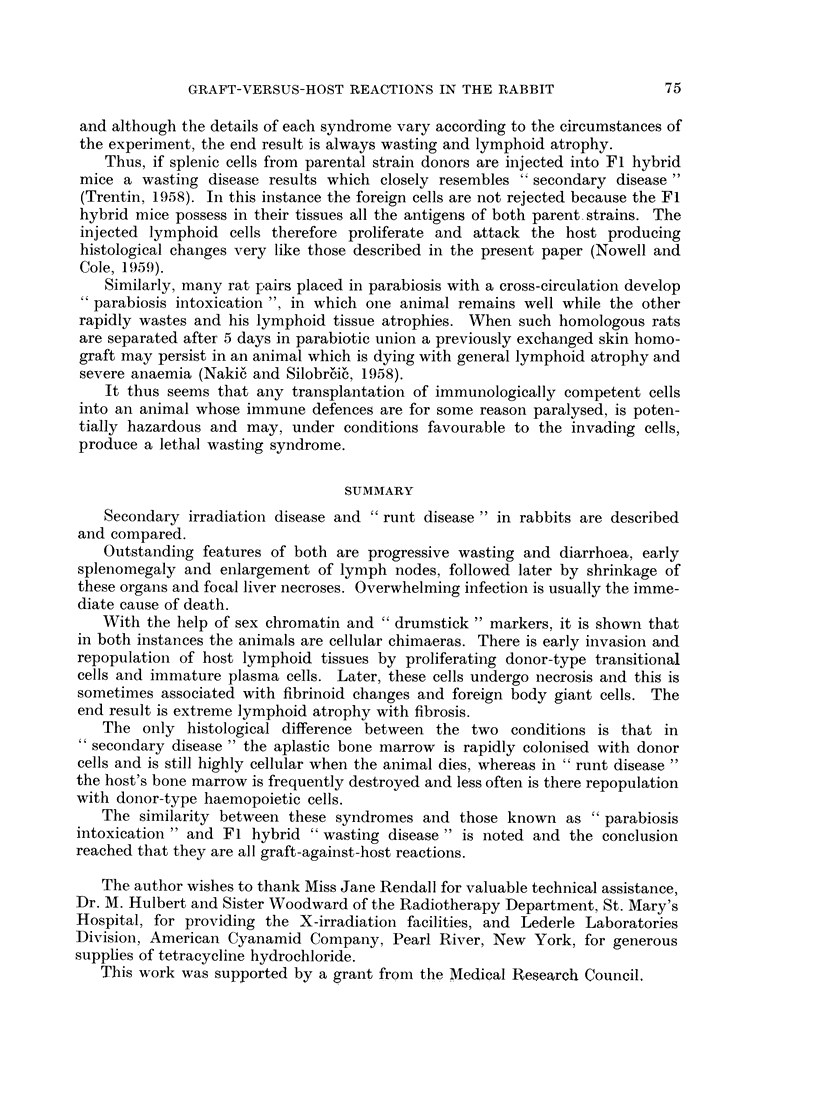

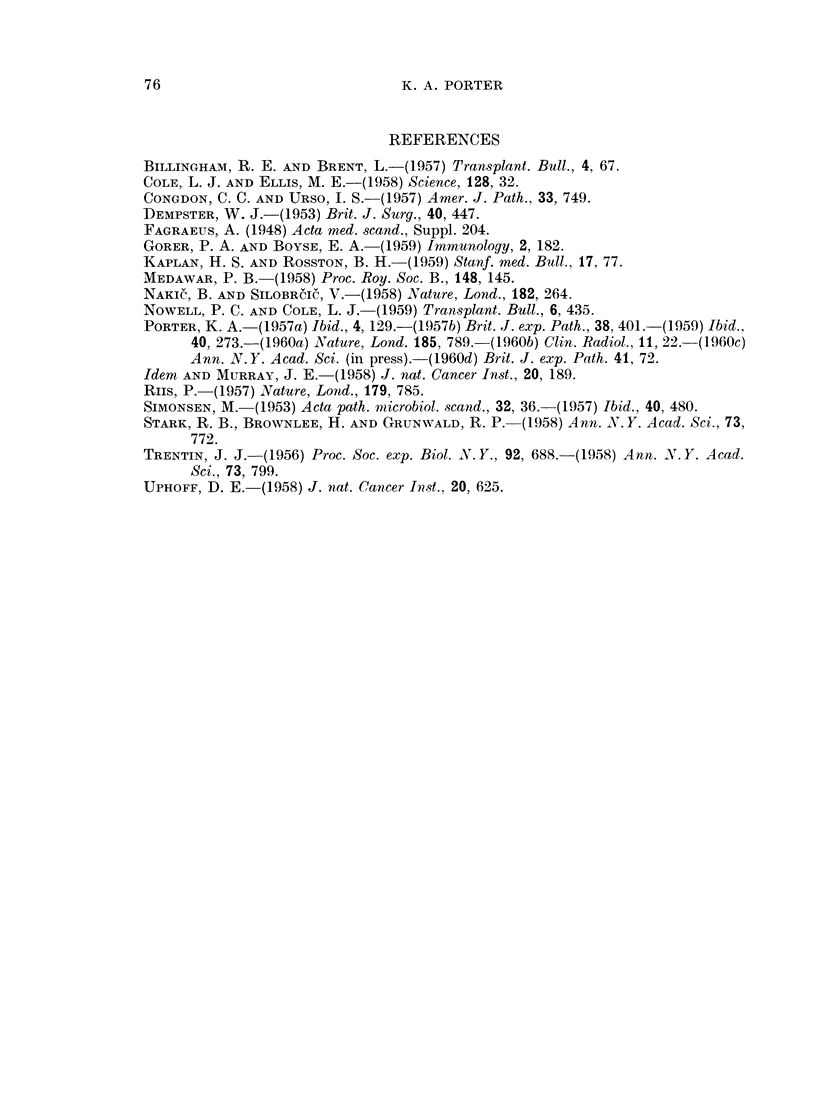


## References

[OCR_00739] CONGDON C. C., URSO I. S. (1957). Homologous bone marrow in the treatment of radiation injury in mice.. Am J Pathol.

[OCR_00740] DEMPSTER W. J. (1953). Kidney homotransplantation.. Br J Surg.

[OCR_00744] GORER P. A., BOYSE E. A. (1959). Pathological changes in F1 hybrid mice following transplantation of spleen cells from donors of the parental strains.. Immunology.

[OCR_00749] NAKIC B., SILOBRCIC V. (1958). Tolerance of skin homografts related to fatal disease in separated rat parabionts.. Nature.

[OCR_00751] NOWELL P. C., COLE L. J. (1959). Pathologic changes in old non-irradiated F1 hybrid mice injected with parental-strain spleen cells.. Transplant Bull.

[OCR_00758] RILS P. (1957). A sex difference in the chromatin structure of the peripheral blood lymphocytes.. Nature.

[OCR_00760] SIMONSEN M. (1957). The impact on the developing embryo and newborn animal of adult homologous cells.. Acta Pathol Microbiol Scand.

[OCR_00762] STARK R. B., BROWNLEE H., GRUNWALD R. P. (1958). Homologous whole blood as an agent for enhancement of skin grafts in the adult rabbit: a preliminary report.. Ann N Y Acad Sci.

[OCR_00766] TRENTIN J. J. (1958). Tolerance and homologous disease in irradiated mice protected with homologous bone marrow.. Ann N Y Acad Sci.

[OCR_00770] UPHOFF D. E. (1958). Perclusion of secondary phase of irradiation syndrome by inoculation of fetal hematopoietic tissue following lethal total-body x-irradiation.. J Natl Cancer Inst.

